# A prediction model for functional outcome following rescue therapy in patients with acute ischemic stroke undergoing endovascular treatment

**DOI:** 10.1080/07853890.2026.2719889

**Published:** 2026-08-19

**Authors:** Xiaoyu Wu, Lanqi Li, Mingchen Zhang, Kangjia Song, Wenbin Zhang, Siyuan Wang, Chao Jiang, Shouchun Wang

**Affiliations:** a Stroke Center, Department of Neurology, The First Hospital of Jilin University, Changchun, China; b Department of Neurology, Affiliated Hospital of Beihua University, Jilin, China; c Department of Neurology, Northern Jiangsu People’s Hospital Affiliated to Yangzhou University, Yangzhou, China

**Keywords:** Acute ischemic stroke, endovascular treatment, rescue therapy, prognostic model

## Abstract

**Objective:**

To evaluate the association between key preoperative and intraoperative factors and 90-day functional outcomes in acute ischemic stroke (AIS) patients undergoing endovascular treatment (EVT) with rescue therapy, and to establish a prognostic model for estimating 90-day functional outcomes.

**Methods:**

We retrospectively enrolled AIS patients with EVT plus rescue therapy from January 2018 to December 2024. The primary endpoint was a 90-day modified Rankin Scale (mRS) score >2. Logistic regression identified predictors incorporated into a nomogram. Model performance was assessed with receiver operating characteristic (ROC) curves, the Hosmer–Lemeshow test, and decision curve analysis (DCA). Bootstrap resampling and 10-fold cross-validation assessed robustness.

**Results:**

In total, 445 individuals were analyzed (training: 312; validation: 133). Multivariable analysis identified glycated hemoglobin (HbA1c; OR = 1.345, 95% CI: 1.145–1.608, *p* < 0.001), baseline National Institutes of Health Stroke Scale (NIHSS) score (OR = 1.084, 95% CI: 1.047–1.127, *p* < 0.001), onset-to-puncture time (OPT; OR = 1.000, 95% CI: 1.000–1.000, *p* = 0.003), and puncture-to-reperfusion time (PRT; OR = 1.008, 95% CI: 1.001–1.015, *p* = 0.026) as independent predictors. The value of the area under the ROC curve (AUC) was 0.741 in the training cohort and 0.781 in the validation cohort. Calibration was adequate (Hosmer–Lemeshow *p* > 0.05 in both cohorts), and DCA demonstrated consistent clinical benefit. Bootstrap and cross-validation confirmed model reliability.

**Conclusion:**

Our model integrating HbA1c, NIHSS, OPT, PRT, and refractory thrombectomy demonstrated acceptable performance in predicting 90-day outcomes after rescue therapy. However, as a development-phase model, its clinical applicability should be interpreted with caution.

## Introduction

Acute ischemic stroke (AIS) remains a major neurological disorder concern, associated with substantial mortality and disability rates, and its burden is expected to rise further with population aging [1,2]. Endovascular treatment (EVT) has emerged as a first-line therapy for patients with large vessel occlusion (LVO), significantly improving early reperfusion rates and functional outcomes in selected patients, while also driving advances in acute stroke care systems [3,4]. However, in patients with atherosclerotic lesions, thrombectomy may still result in recanalization failure or residual high-grade stenosis, leading to inadequate or unstable intracranial perfusion [5]. For such cases, rescue therapy – including balloon angioplasty alone or stent implantation – has become an essential strategy to achieve successful vessel recanalization and is now widely implemented in clinical practice [6]. Previous studies have demonstrated that rescue therapy enhances functional outcomes and recanalization rates in comparison to medical management alone [7–10].

Nevertheless, a recent multicenter randomized controlled trial (ANGEL-REBOOT) reported no significant improvement in 90-day functional outcome with rescue therapy [11]. These findings indicate that even when satisfactory recanalization is achieved, functional recovery can vary among individuals, which can likely be attributed to the interplay of clinical and procedural factors. At present, no predictive tools are available to estimate functional outcomes in patients undergoing rescue therapy, and risk assessment largely relies on operator experience, limiting precision in clinical judgment and individualized decision-making.

Based on these considerations, we aimed to systematically evaluate the relationship of pre- and intraoperative clinical characteristics with 90-day functional outcomes among individuals with AIS undergoing rescue interventions, and further develop and validate a visual prediction model to provide clinicians with quantitative risk assessment.

## Methods

### Study design

**Figure 1.**
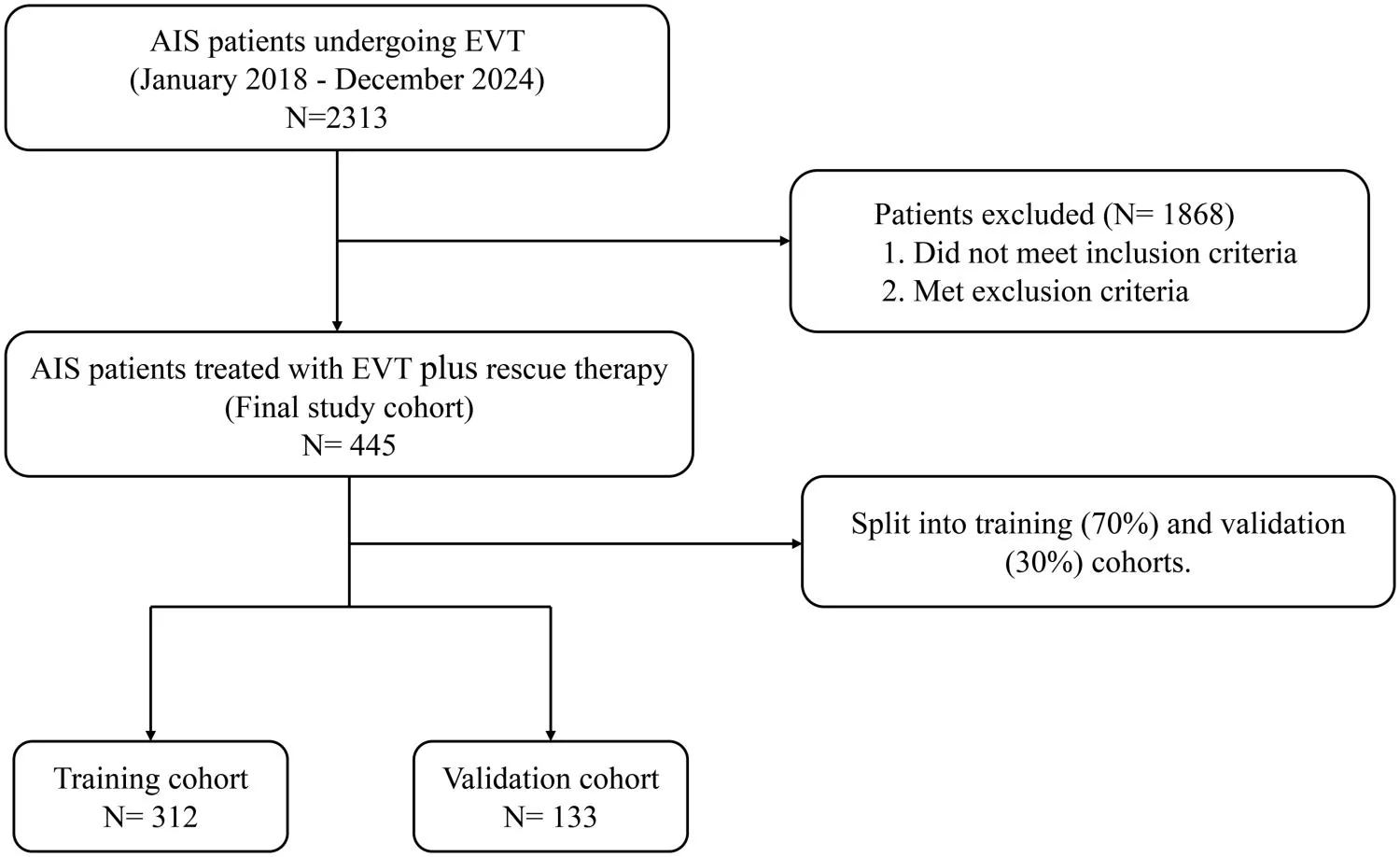
Flowchart of Patient Selection and Cohort Allocation. AIS, acute ischemic stroke; EVT, endovascular treatment.

Patients with AIS who received EVT between January 2018 and December 2024 at a comprehensive stroke center were retrospectively analyzed in this single-center study. Inclusion criteria were as follows: (1) age ≥18 years, any sex; (2) pre-stroke modified Rankin Scale (mRS) score of 0–1; (3) receipt of rescue therapy (balloon angioplasty alone or stent implantation) during emergent EVT; (4) target lesion located in the intracranial artery segment (the intracranial segment of the internal carotid artery, the M1 portion of the middle cerebral artery, the junction of the vertebral and basilar arteries, or the basilar artery trunk); (5) post-procedural expanded Thrombolysis in Cerebral Infarction (eTICI) score >2B/3. Patients were excluded if they met any of the following criteria: (1) presence of tandem intracranial-extracranial lesions; (2) severe cardiopulmonary disease or hepatic/renal failure; (3) malignancy or other severe conditions with a life expectancy of <90 days; (4) patients lacking essential clinical data or those not successfully followed up (Figure 1).

Ethics Statement:

This study was approved by the Ethics Committee of the First Hospital of Jilin University (Approval No. 23K177-002). Given its retrospective design and use of anonymized clinical data, the requirement for informed consent was waived in accordance with institutional and national regulations.

### Data collection

Patient information was sourced from the hospital’s digital medical record database and consisted of the following items: (1) general demographics and medical history such as sex, age, hypertension, diabetes, coronary artery disease, atrial fibrillation, hyperlipidemia, prior stroke, smoking history, alcohol consumption, and other relevant factors; (2) baseline characteristics including systolic and diastolic blood pressure, National Institutes of Health Stroke Scale (NIHSS) score, and other related parameters; (3) imaging data including Alberta Stroke program Early CT Score (ASPECTS) score and other imaging indicators; (4) laboratory data comprising blood glucose level at admission, glycated hemoglobin (HbA1c), and additional laboratory tests; (5) time metrics including onset-to-admission time, onset-to-puncture time, puncture-to-reperfusion time, admission-to-puncture time, and other time-related variables; and (6) procedural details including anesthesia modality, lesion location, first-choice thrombectomy technique, type of rescue therapy, refractory thrombectomy, procedure-related complications, and other procedural variables. Refractory thrombectomy was defined as a total of three or more thrombectomy passes performed using stent retrievers or aspiration devices [12,13].

### Definition of rescue therapy

Rescue therapy was defined using intraoperative digital subtraction angiography assessment. Patients who did not achieve successful recanalization after conventional thrombectomy (eTICI <2b) or who had significant residual stenosis (≥70%) following thrombectomy underwent either balloon angioplasty alone or stent implantation. Patients who initially received balloon angioplasty but developed a high risk of re-occlusion due to severe vessel dissection or recoil were subsequently treated with stent implantation and classified under the stent-based rescue therapy group.

### Follow-up and endpoints

At 3 months post-procedure, patients were evaluated either at outpatient clinics or *via* telephone, during which experienced neurologists assessed the mRS score. Outcome assessment was performed without reference to patients’ baseline and procedural information. Unfavorable functional outcome at 3 months, specified as an mRS score above 2, was considered the primary study endpoint.

### Statistical analysis

Normality of continuous variables was assessed with the Shapiro–Wilk test. Variables with normal distribution were reported as mean ± standard deviation (Mean ± SD) and compared using the independent-samples t-test, whereas non-normally distributed variables were summarized as median with interquartile range (M [Q1, Q3]) and compared using the Mann–Whitney U test. Categorical variables were expressed as numbers with percentages (n, %) and compared using the chi-square or Fisher’s exact test, as appropriate. Missing data were assessed prior to analysis. Specifically, ASPECTS was missing in 8 patients and admission glucose was missing in 2 patients. Multiple imputation was performed to handle missing values, and the imputed datasets were used to generate a complete dataset for subsequent analyses. The dataset was subsequently split into training (70%) and validation (30%) sets. Candidate predictors identified with *p* < 0.1 in univariate logistic regression were further entered into the multivariate logistic regression framework. A backward stepwise selection based on the likelihood ratio (LR) was then performed using SPSS, with a removal criterion of *p* > 0.10. The discriminatory ability, calibration quality, and clinical applicability of the model were assessed through three analytical approaches: receiver operating characteristic (ROC) curve analysis, Hosmer–Lemeshow goodness-of-fit test, and decision curve analysis (DCA). To further validate model stability, 1000 bootstrap resamples and 10-fold cross-validation were performed. In a sensitivity analysis, thrombectomy year was incorporated into the multivariable logistic regression model as a continuous variable to assess whether temporal changes in thrombectomy practice during the study period influenced model performance. Differences in model discrimination between models with and without thrombectomy year were compared using the DeLong test. All statistical analyses were conducted using SPSS Statistics (version 29.0.1.0, IBM) and R (version 4.4.1, R Foundation). A two-sided *p* < 0.05 was adopted as the significance threshold.

## Results

### Baseline characteristics

A total of 445 patients were included in the study. Stratified random sampling was performed according to prognostic type and type of rescue therapy, and the baseline characteristics were well-balanced across the training and validation sets (Supplemental Table 1). The training set comprised 312 patients, of whom 244 were males (78.2%) with a median age of 60 (52–67) years. Among these, 179 patients (57.4%) experienced unfavorable outcomes. An initial univariate analysis indicated that the unfavorable outcome group had a lower proportion of males (73.9% vs. 84.1%, *p* = 0.033), a higher prevalence of diabetes mellitus (33.9% vs. 20.5%, *p* = 0.010) and coronary artery disease (17.8% vs. 8.3%, *p* = 0.019), higher admission blood glucose levels (7.9 vs. 7.2 mmol/L, *p* = 0.012), higher baseline NIHSS scores (13.5 vs. 11.0, *p* < 0.001), and elevated HbA1c levels (6.2 vs. 5.7%, *p* < 0.001), among other variables (Table 1).

**Table 1.** Univariate analysis of clinical characteristics between patients with favorable and unfavorable outcomes in the training cohort.

Variables	Total (*N* = 312)	Favorable outcome (*N* = 132)	Unfavorable outcome (*N* = 180)	P value
Male, n (%)	244(78.2)	111(84.1)	133(73.9)	0.033**
Age, M (Q1, Q3), years	60.0(52.0-67.0)	59.5(50.5-66.0)	61.0(53.5-67.0)	0.080*
Medical history				
HT, n (%)	206(66.0)	89(67.4)	117(65.0)	0.655
DM, n (%)	88(28.2)	27(20.5)	61(33.9)	0.010**
AF, n (%)	8(2.6)	1(0.8)	7(3.9)	0.121
CHD, n (%)	43(13.8)	11(8.3)	32(17.8)	0.019**
Hyperlipidemia, n (%)	142(45.5)	55(41.7)	87(48.3)	0.243
Prior stroke, n (%)	98(31.4)	43(32.6)	55(30.6)	0.704
Wake-up stroke, n (%)	64(20.5)	29(22.0)	35(19.4)	0.585
Past or current smoking, n (%)	154(49.4)	70(53)	84(46.7)	0.267
Past or current drinking, n (%)	110(35.3)	49(37.1)	61(33.9)	0.555
Baseline data				
SBP, M (Q1, Q3), mmHg	154.0(141.0-173.0)	153.0(140.5-173.0)	156.5(141.0-173.5)	0.521
DBP, M (Q1, Q3), mmHg	92.0(81.5-100.0)	90.5(82.0-99.0)	92.0(80.5-100.0)	0.558
MAP, M (Q1, Q3), mmHg	111.7(102.0-123.9)	111.2(102.0-122.7)	112.3(102.0-124.7)	0.497
GLU, M (Q1, Q3), mmol/L	7.5(6.3-9.4)	7.2(6.1-8.4)	7.9(6.5-10.0)	0.012**
NIHSS, M (Q1, Q3)	12.0(9.0-16.0)	11.0(8.0-14.5)	13.5(11.0-18.0)	<0.001**
ASPECTS, M (Q1, Q3)	8.0(7.0-9.0)	8.0(7.0-10.0)	8.0(7.0-9.0)	0.278
Procedural variables				
ODT, M (Q1, Q3), min	325.5(177.5-746.0)	307.5(160.5-630.0)	339.0(180.0-852.5)	0.042**
OPT, M (Q1, Q3), min	488.0(344.5-1027.5)	465.0(349.5-862.5)	530.0(340.5-1115.0)	0.041**
PRT, M (Q1, Q3), min	80.0(60.0-110.0)	75.0(52.0-99.0)	90.0(62.0-118.5)	0.003**
DPT, M (Q1, Q3), min	135.0(100.0-177.5)	140.5(100.0-175.0)	126.0(99.5-178.0)	0.756
TOAST classification				0.877
LAA, n (%)	282(90.4)	120(90.9)	162(90.0)	
CE, n (%)	8(2.6)	2(1.5)	6(3.3)	
Others, n (%)	22(7.1)	10(7.6)	12(6.7)	
Occlusion site,				0.697
Basilar artery, n (%)	79(25.3)	35(26.5)	44(24.4)	
Vertebrobasilar junction n (%)	66(21.2)	27(20.5)	39(21.7)	
ICA-intracranial segment n (%)	52(16.7)	15(11.4)	37(20.6)	
Middle cerebral artery n (%)	115(36.9)	55(41.7)	60(33.3)	
First-line thrombectomy				0.967
Stent-retriever, n (%)	6(1.9)	2(1.5)	4(2.2)	
Aspiration, n (%)	27(8.7)	11(8.3)	16(8.9)	
Stent-retriever plus aspiration, n (%)	279(89.4)	119(90.2)	160(88.9)	
Anesthesia				0.029**
General Anesthesia, n (%)	199(63.8)	75(56.8)	124(68.9)	
Local Anesthesia, n (%)	94(30.1)	48(36.4)	46(25.6)	
Sedation, n (%)	19(6.1)	9(6.8)	10(5.6)	
IVT				0.950
rt-PA, n (%)	34(10.9)	16(12.1)	18(10.0)	
TNK, n (%)	6(1.9)	4(2.2)	2(1.5)	
UK, n (%)	3(1.0)	2(1.1)	1(0.8)	
Types of Circulation				0.881
Anterior Circulation, n (%)	167(53.5)	70(53.0)	97(53.9)	
Posterior Circulation, n (%)	145(46.5)	62(47.0)	83(46.1)	
Way of rescue				0.178
Balloon, n (%)	214(68.6)	96(72.7)	118(65.6)	
Stent, n (%)	98(31.4)	36(27.3)	62(34.4)	
HbA1c, n (%), %	5.9(5.5-7.1)	5.7(5.4-6.3)	6.2(5.6-7.9)	<0.001**
Tirofiban treatment, n (%)	256(82.1)	113(85.6)	143(79.4)	0.163
Refractory_thrombectomy, n (%)	51(16.3)	14(10.6)	37(20.6)	0.021**
Vessel dissection, n (%)	21(6.7)	10(7.6)	11(6.1)	0.611
Vascular perforation, n (%)	2(0.6)	0(0.0)	2(1.1)	0.999
Distal embolization, n (%)	21(6.7)	6(4.5)	15(8.3)	0.194

*p < 0.1.

**p < 0.05.

HT, hypertension; DM, diabetes mellitus; AF, atrial fibrillation; CHD, coronary heart disease; SBP, systolic blood pressure; DBP, diastolic blood pressure; MAP, mean arterial pressure; GLU, glucose; NIHSS, National Institutes of Health Stroke Scale; ASPECTS, Alberta Stroke Program Early CT Score; ODT, Onset-to-door time; OPT, Onset-to-puncture time; PRT, Puncture-to-recanalization time; DPT, Door-to-puncture time; LAA, large-artery atherosclerosis; CE, cardioembolism; ICA, internal carotid artery; rt-PA, recombinant tissue-type plasminogen activator; TNK, tenecteplase; UK, urokinase; HbA1c, glycated hemoglobin; Refractory thrombectomy, Number of passes ≥ 3 times.

### Model development and evaluation

This model comprised five variables, with results indicating that HbA1c (HbA1c; OR = 1.345, 95% CI: 1.145–1.608, *p* < 0.001), baseline NIHSS score (OR = 1.084, 95% CI: 1.047–1.127, *p* < 0.001), puncture-to-reperfusion time (PRT; OR = 1.008, 95% CI: 1.001–1.015, *p* = 0.026), and onset-to-puncture time (OPT; OR = 1.000, 95% CI: 1.000–1.000, *p* = 0.003) were independent predictors of unfavorable outcomes (Table 2). The full logistic regression model with full-precision coefficients, including the intercept term, is provided in Supplemental Table 2.

**Table 2.** Multivariate logistic regression analysis of predictors selected from univariate analysis.

Variables	β	OR (95% CI)	P value
HbA1c	0.297	1.345(1.145–1.608)	<0.001
NIHSS	0.081	1.084(1.047–1.127)	<0.001
OPT	0.000	1.000(1.000-1.000)	0.003
PRT	0.008	1.008(1.001–1.015)	0.026
Refractory thrombectomy	0.642	1.901(0.915–4.089)	0.091

OR, odds ratio; CI, confidence interval.

HbA1c, glycated hemoglobin; NIHSS, National Institutes of Health Stroke Scale; OPT, Onset-to-puncture time; PRT, Puncture-to-recanalization time; Refractory thrombectomy, Number of passes ≥ 3 times.

**Figure 2.**
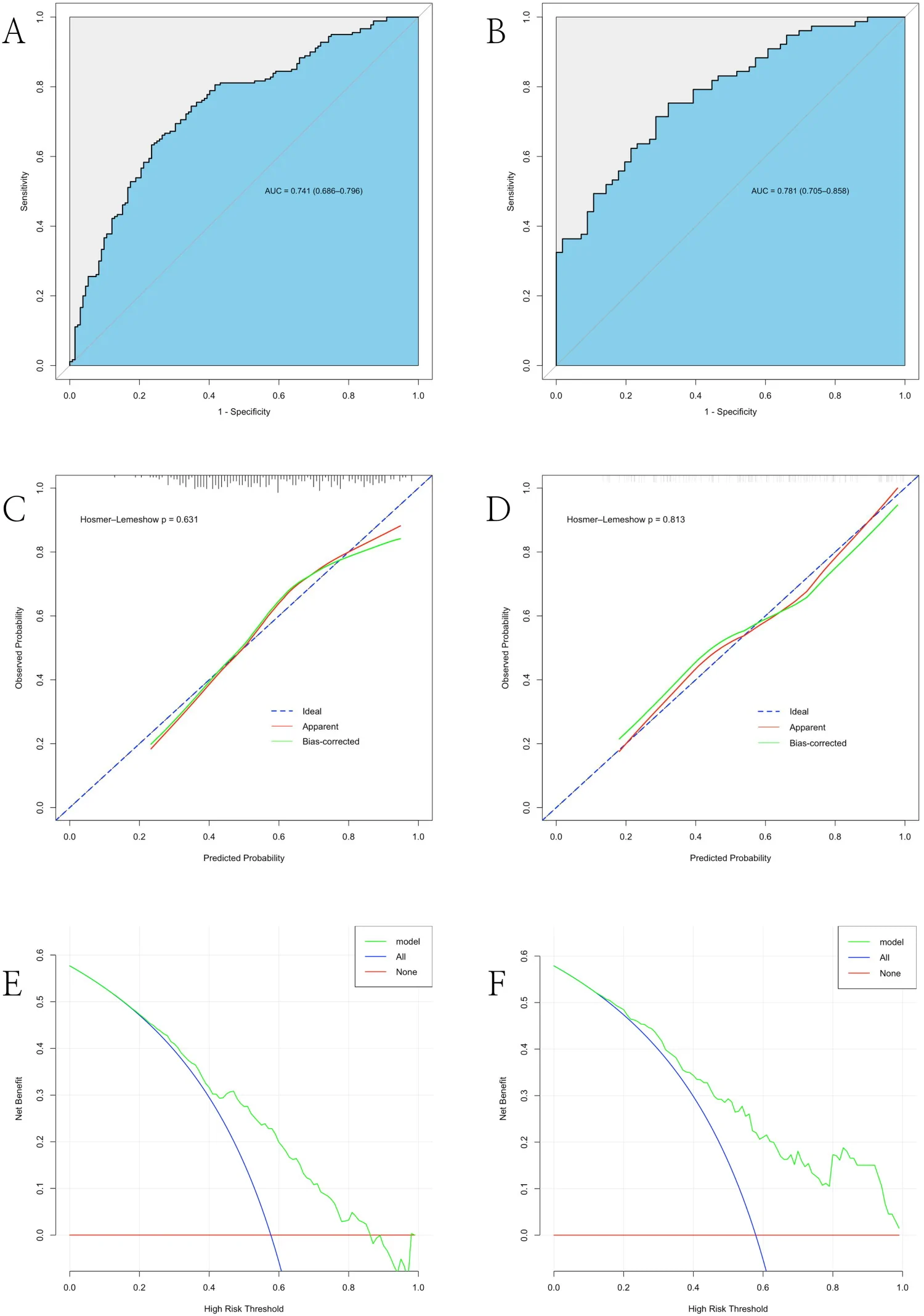
Model performance in the training (A, C, E) and validation (B, D, F) cohorts. Receiver operating characteristic (ROC) curves (A, B), Hosmer–Lemeshow (HL) calibration curves (C, D), and decision curve analyses (DCA) (E, F).

The area under the receiver operating characteristic curve (AUC) was 0.741 (95% CI: 0.686–0.796) for the training set and 0.781 (95% CI: 0.705–0.858) for the validation set, indicating good discrimination (Figure 2(A,B)). In the Hosmer–Lemeshow test, P values were 0.631 for the training set and 0.813 for the validation set, both >0.05, further indicating good concordance between model predictions and observed outcomes. Moreover, the calibration plot with 1,000 bootstrap resamples showed that the bias-corrected curve closely overlapped with the apparent curve, suggesting robust internal consistency. (Figure 2(C,D)). Quantitative calibration analysis showed that the calibration slope and intercept were 1.00 and 0.00 in the training set, respectively. In the validation set, the calibration slope was 1.55, while the intercept was 0.10, indicating a deviation from perfect calibration in the scaling of predicted risks, with no substantial systematic over- or underestimation at the population level. The Brier score was 0.203 in the training set and 0.192 in the validation set, suggesting acceptable overall predictive performance. DCA revealed positive net clinical benefit across the training and validation sets, outperforming the ‘treat-all’ and ‘treat-none’ strategies, thus indicating promising clinical applicability (Figure 2(E,F)). Additionally, 10-fold cross-validation yielded stable AUC values, as supporting the reproducibility of the model (Supplemental Figure 1). Bootstrap resampling yielded an optimism-corrected AUC of 0.727, with a small optimism estimate of 0.014, indicating minimal overfitting.

### Visualization of the predictive model

**Figure 3.**
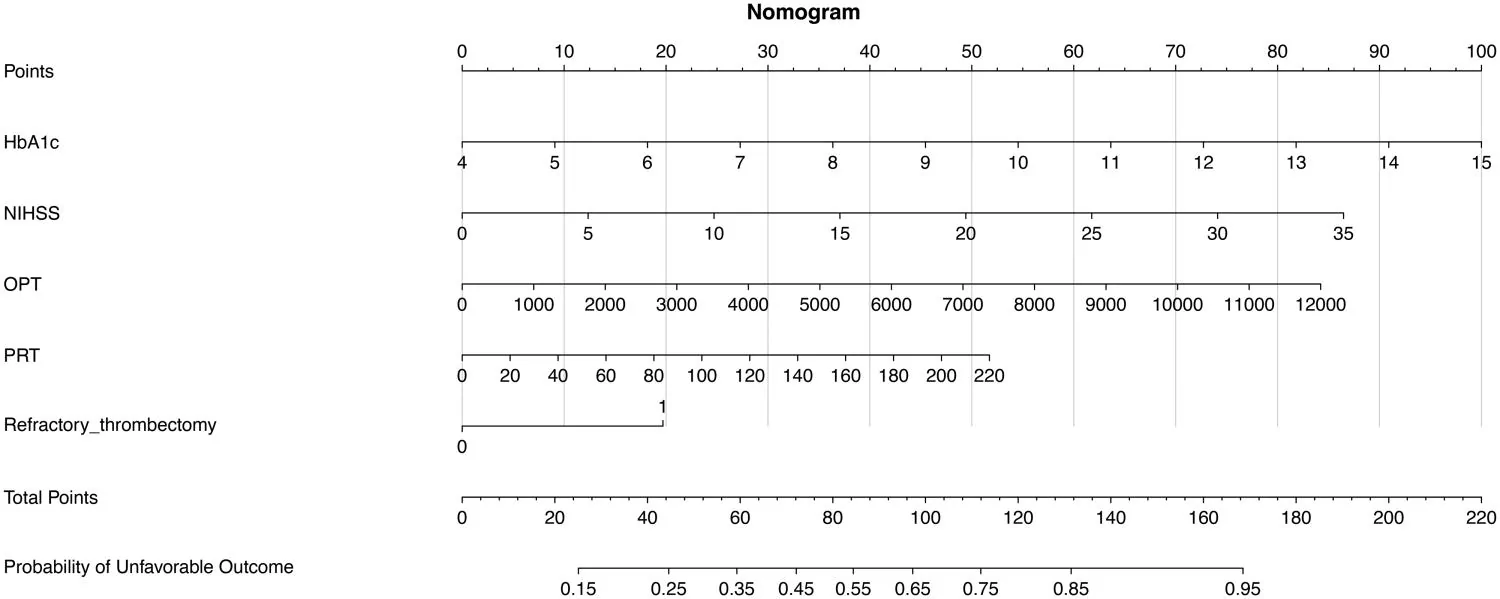
Nomogram for predicting 90-day unfavorable functional outcome in acute ischemic stroke patients after rescue endovascular therapy. Each variable value corresponds vertically to its point on the nomogram, and the total points correspond vertically to the predicted probability of poor functional outcome. HbA1c, glycated hemoglobin; NIHSS, National Institutes of Health Stroke Scale; OPT, Onset-to-puncture time; PRT, Puncture-to-recanalization time; Refractory thrombectomy, Number of passes ≥ 3 times.

To facilitate individualized prediction of unfavorable outcomes in AIS patients who received endovascular rescue therapy, a nomogram was constructed using multivariate logistic regression analysis (Figure 3). The nomogram assigns specific scores to each value of the five variables, and the cumulative score corresponds to the predicted probability of an unfavorable outcome for each patient. An enlarged view focusing on the clinically relevant ranges of OPT and PRT is also provided to facilitate point reading (Supplemental Figure 2).

To ensure transparency and reproducibility, the full logistic regression equation with full-precision coefficients, including the intercept term, is provided as follows:

$$\begin{array}{c} \text{logit }\left(P\right)=-3.739+0.2965*\text{HbA1c }+0.0806*\text{NIHSS }+0.000229*\text{OPT}\\ +0.007669*\text{PRT}+0.6425*\text{Refractory thrombectomy} \end{array}$$

where P represents the probability of an unfavorable functional outcome. The predicted probability was calculated using the logistic (inverse logit) function:

$$P=1/(1+e^{(-\text{logit}(}P)))$$

### Sensitivity analysis

To assess the potential influence of temporal changes, thrombectomy year was incorporated into the multivariable logistic regression model as a continuous variable. Thrombectomy year was not independently associated with 90-day functional outcome (OR 1.097, 95% CI 0.941–1.283, *p* = 0.240). The inclusion of thrombectomy year did not significantly improve model discrimination in either the training cohort (AUC 0.741 vs. 0.742, *p* = 0.912) or the validation cohort (AUC 0.781 vs. 0.766, *p* = 0.552) (Supplemental Tables 3–4).

## Discussion

This study is the first to present a validated prognostic model in AIS patients undergoing endovascular rescue therapy following emergent thrombectomy. Using real-world data, we developed and validated a predictive model associated with 90-day functional outcomes. The model integrates five key variables: HbA1c, baseline NIHSS score, OPT, PRT, and refractory thrombectomy. The predictive accuracy of the model was confirmed in both the training dataset and the independent validation cohort. Furthermore, when integrated with a nomogram, individualized risk assessment can be rapidly performed, providing clinicians with quantitative guidance for perioperative risk assessment and highlighting its potential clinical applicability and translational value.

HbA1c, a marker of long-term glycemic control, showed significant correlation with unfavorable 90-day functional outcomes (OR 1.345, 95% CI 1.145–1.608, *p* < 0.001). The potential mechanisms may include: (1) elevated levels of advanced glycation end-products (AGEs), which enhance oxidative stress and cause vascular endothelial injury [14]; (2) chronic hyperglycemia combined with acute hypoxia, which enhances inflammation and a prothrombotic state, thereby aggravating microcirculatory hypoperfusion [15–17]; (3) induction of lipid metabolism disorders, with elevated LDL exerting synergistic detrimental effects on ischemic stroke [18]. Although baseline admission blood glucose differed significantly between the favorable and unfavorable outcome groups in univariate analysis (7.2 vs. 7.9 mmol/L, *p* = 0.012), this was not included in the final predictive model, possibly because HbA1c more reliably reflects long-term metabolic status. This indicates that poor long-term glycemic control may have a more profound impact on functional recovery after rescue therapy. In addition to these metabolic mechanisms, elevated HbA1c may also reflect vascular alterations associated with chronic dysglycemia, including intracranial atherosclerotic disease (ICAD). Although large artery atherosclerosis (LAA) was highly prevalent according to the TOAST classification in our cohort and its distribution did not differ significantly between patients with favorable and unfavorable outcomes, TOAST classification is primarily designed for stroke etiological categorization and may not fully capture the specific imaging characteristics of intracranial vascular lesions or the associated procedural complexity. In patients undergoing EVT who subsequently require rescue therapy, diabetes-related intracranial atherosclerotic changes may reflect more severe vascular pathology, increasing the risk of residual stenosis after initial EVT and re-occlusion, thereby potentially affecting subsequent functional recovery. These findings are consistent with previous studies linking abnormal glucose metabolism to adverse stroke outcomes [19–21], and they offer further guidance for perioperative glycemic management and secondary prevention strategies.

Baseline NIHSS score was also associated with unfavorable 90-day functional outcomes following rescue therapy (OR 1.084, 95% CI 1.047–1.127, *p* < 0.001), reflecting the extent and severity of neurological impairment present at admission. In a large-scale study, Prof. Reznik and colleagues reported that, compared with the predictive ability of baseline NIHSS for 90-day functional outcomes (AUC 0.635, 95% CI 0.566–0.704), NIHSS scores at 24 h post-procedure and at discharge may perform better (AUC 0.846, 95% CI 0.798–0.895; AUC 0.873, 95% CI 0.832–0.914) [22]. However, this study aimed to develop a model integrating preoperative and intraoperative clinical features, emphasizing prognostic assessment prior to the decision for rescue therapy. As a preoperative indicator, baseline NIHSS remains closely associated with functional outcomes [23]. Based on these considerations, we validated that baseline NIHSS maintains stable predictive ability in a highly heterogeneous population undergoing rescue therapy, thereby assisting clinicians in preliminary prognostic trends and adjustment of treatment strategies.

In this predictive model, time-related variables had a significant impact on postoperative functional outcomes. Both OPT and PRT were independent predictors (*p* = 0.003 and *p* = 0.026, respectively), further supporting the ‘time is brain’ concept in AIS management [24]. OPT primarily reflects the overall efficiency of prehospital stroke recognition, patient transport, and in-hospital preoperative workflow. In our training cohort, the median OPT was 488 min (interquartile range: 344.5–1027.5 min). This pattern likely reflects the role of our center as a national advanced stroke center within a regional referral network, receiving patients transferred from across the province and neighboring areas, which may lead to prolonged prehospital delays. Despite these interregional transfer-related delays, evidence from studies such as DAWN and DEFUSE 3 indicates that EVT remains a crucial treatment strategy following comprehensive evaluation [25–27]. PRT reflects the efficiency of intraoperative reperfusion and the complexity of the vascular lesion. Although the odds ratios for OPT and PRT were close to 1 (OR 1.000, 95% CI 1.000–1.000; OR 1.008, 95% CI 1.001–1.015), cumulative delays measured in hours may substantially increase the risk of unfavorable outcomes, consistent with previous reports [28,29]. Based on the model coefficient, a 60-minute increase in OPT was associated with an approximately 1.4% increase in the odds of unfavorable outcome. A similar pattern was observed for PRT, further reinforcing the stable association between treatment delays and poor prognosis.

Additionally, refractory thrombectomy was included in our model with a P value of 0.091. Although it did not reach statistical significance, it was retained during stepwise selection, suggesting that it may serve as an indirect indicator of the functional cost associated with repeated intraoperative thrombectomy and time delays. A sensitivity analysis excluding refractory thrombectomy showed a slight decrease in model discrimination, suggesting a modest but consistent contribution of this variable to the overall predictive performance and supporting its retention in the final model. Anesthesia modality was also associated with outcome in univariate analysis but was not retained in the final model. This likely reflects the fact that anesthesia choice is often influenced by patient condition and procedural difficulty, which are closely related to baseline NIHSS and procedure-related time. After accounting for these factors, the association between anesthesia modality and outcome was attenuated.

Overall, the present model estimates the risk of unfavorable functional outcomes in patients undergoing rescue therapy and distinguishes between patients with relatively higher and lower baseline risk. In this context, it is important to consider evidence from trials such as ANGEL-REBOOT [11], which did not demonstrate an overall benefit of rescue therapy. The model is not intended to determine whether rescue therapy should be performed, but rather to describe the risk profile of patients managed under current clinical practice. By characterizing differences in baseline risk, the model may help to better understand the relationship between procedural burden and long-term functional outcomes, particularly in situations where the overall benefit of rescue therapy remains uncertain. However, the model does not estimate treatment effect and should not be used to guide treatment decisions at the individual level. It should be acknowledged that key variables in this model, particularly baseline NIHSS and time-related metrics, are already integral to routine stroke assessment. Rather than introducing new predictors, this model combines these commonly used factors within the context of rescue therapy, allowing their joint effects to be quantified. In this context, it lays a foundation for future multicenter prospective studies on rescue therapy and the development of clinical prediction models.

Beyond the interpretation of the model findings, its development and validation strategy also warrants consideration. Stratified random sampling was adopted to ensure balanced distributions of outcome status and rescue therapy types between the training and validation sets, thereby improving comparability between cohorts. Within this validation framework, the model showed good discrimination and acceptable overall predictive performance, which was further supported by the Brier score. However, calibration analysis revealed an additional point that merits attention: the calibration slope in the validation cohort was 1.55, suggesting that the model tended to produce relatively conservative probability estimates, with a compressed gradient of predicted risk across individuals, while the calibration intercept remained close to zero, indicating accurate estimation of the average event risk at the population level. This finding suggests that, within the current single-center validation setting, the model may be more suitable for broad risk stratification or group-level risk estimation, while interpretation of predicted probabilities at the individual level should be approached with caution. Furthermore, given the relatively long study enrollment period, we performed a sensitivity analysis by incorporating the year of thrombectomy into the multivariable model. The results showed that the year of thrombectomy was not an independent predictor of functional outcome, and its inclusion did not significantly improve the discriminative performance of the model. Nevertheless, future studies incorporating validation strategies across different temporal periods are warranted to further evaluate the model’s stability and generalizability across evolving clinical settings.

## Limitations

This study had certain limitations. First, the analysis was based on retrospective data collected from one medical center, which may have led to unavoidable selection bias. Second, key imaging variables, such as core infarct volume and perfusion mismatch ratio, were excluded due to data unavailability, which may limit the upper bound of the model’s discriminative performance. In contrast, missingness among variables included in the analysis was minimal and handled using imputation, and is therefore unlikely to have materially influenced the model estimates. Third, although time-related variables (OPT and PRT) were statistically significant, they may have been influenced by preoperative workflow and intraoperative procedures; therefore, their generalizability across different centers requires further validation. Fourth, the number of patients with refractory thrombectomy and those experiencing complications was limited, indicating that subsequent studies will need larger cohorts and data from multiple centers to enhance the model’s predictive performance and external applicability.

## Conclusion

This study developed a prognostic model integrating HbA1c, baseline NIHSS, OPT, PRT, and refractory thrombectomy that may assist in estimating 90-day outcomes following rescue therapy. While the model demonstrated acceptable performance in this real-world cohort, it should still be regarded as a development-phase model. Accordingly, its potential clinical applicability will require further confirmation through external validation in multicenter cohorts.

Funding and Disclosures:This study received no external funding, and no industry sponsorship or financial support was involved in its design, conduct, analysis, or reporting. All authors declare that they have no conflicts of interest related to this submission, including no financial relationships, industry affiliations, personal conflicts, or other competing interests. None of the authors serve as editorial board members of the journal to which this manuscript is submitted.

## Supplementary Material

Supplementary_material_with_clean.docx

## Data Availability

Data are available on reasonable requests by qualified investigators to the corresponding author.
