# Supplementary material for: A prediction model for functional outcome following rescue therapy in patients with acute ischemic stroke undergoing endovascular treatment
Source: Ann Med. 2026 Aug 19;58(1):2719889. doi: 10.1080/07853890.2026.2719889 (PMC13491895; doi:10.1080/07853890.2026.2719889)
Supplement: Supplementary file 1 [file IANN_A_2719889_SM1718.docx]

Supplemental Table 1. Baseline Characteristics of Training and Validation Cohorts After Stratified Random Sampling

| Variables | Total  （N=445） | Training Cohort  （N=312） | Validation Cohort  （N=133） | P value |
| --- | --- | --- | --- | --- |
| Male, n (%) | 345（77.5） | 244（78.2） | 101（75.9） | 0.600 |
| Age, M (Q1, Q3), years | 60（53-67） | 60（52-67） | 60（54-67） | 0.932 |
| Medical history |  |  |  |  |
| HT, n (%) | 300（67.4） | 206（66.0） | 94（70.7） | 0.338 |
| DM, n (%) | 125（28.1） | 88（28.2） | 37（27.8） | 0.934 |
| AF, n (%) | 11（2.5） | 8（2.6） | 3（2.3） | 1.000+ |
| CHD, n (%) | 62（13.9） | 43（13.8） | 19（14.3） | 0.888 |
| Hyperlipidemia, n (%) | 202（45.4） | 142（45.5） | 60（45.1） | 0.938 |
| Prior stroke, n (%) | 148（33.3） | 98（31.4） | 50（37.6） | 0.205 |
| Wake-up stroke, n (%) | 102（22.9） | 64（20.5） | 38（28.6） | 0.064 |
| Past or current smoking, n (%) | 219（49.2） | 154（49.4） | 65（48.9） | 0.925 |
| Past or current drinking, n (%) | 166（37.3） | 110（35.3） | 56（42.1） | 0.171 |
| Baseline data |  |  |  |  |
| SBP, M (Q1, Q3) , mmHg | 155.0（140.0-173.0） | 154.0（141.0-173.0） | 156.0（139.5-171.5） | 0.917 |
| DBP, M (Q1, Q3) , mmHg | 90.0（80.0-99.0） | 92.0（81.3-100.0） | 89.0（79.5-98.0） | 0.076 |
| MAP, M (Q1, Q3) , mmHg | 111.3（102.0-122.7） | 111.7（102.0-123.9） | 110.3（100.8-121.0） | 0.239 |
| GLU, M (Q1, Q3) , mmol/L | 7.3（6.2-9.4） | 7.5（6.3-9.4） | 7.1（6.0-9.5） | 0.201 |
| NIHSS, M (Q1, Q3) | 13.0（9.0-17.0） | 12.0（9.0-16.0） | 13.0（9.0-18.0） | 0.370 |
| ASPECTS, M (Q1, Q3) | 8.0（7.0-9.0） | 8.0（7.0-9.0） | 8.0（7.0-9.0） | 0.765 |
| Procedural variables |  |  |  |  |
| ODT, M (Q1, Q3), min | 330.0（177.5-731.5） | 325.5（176.3-748.0） | 363.0（177.5-637.5） | 0.905 |
| OPT, M (Q1, Q3), min | 510.0（344.5-967.5） | 488.0（344.3-1028.8） | 544.0（344.0-859.0） | 0.848 |
| PRT, M (Q1, Q3), min | 80.0（56.5-110.0） | 80.0（60.0-110.0） | 71.0（54.5-105.5） | 0.143 |
| DPT, M (Q1, Q3), min | 135.0（100.0-179.5） | 135.0（100.0-177.8） | 135.0（100.0-187.5） | 0.936 |
| TOAST classification |  |  |  | 0.968 |
| LAA, n (%) | 402（90.3） | 282（90.4） | 120（90.2） |  |
| CE, n (%) | 11（2.5） | 8（2.6） | 3（2.3） |  |
| Others, n (%) | 32（7.2） | 22（7.1） | 10（7.5） |  |
| Occlusion site, |  |  |  | 0.258 |
| Basilar artery, n (%) | 124（27.9） | 79（25.3） | 45（33.8） |  |
| Vertebrobasilar junction n (%) | 87（19.6） | 66（21.2） | 21（15.8） |  |
| ICA-intracranial segment n (%) | 74（16.6） | 52（16.7） | 22（16.5） |  |
| Middle cerebral artery n (%) | 160（36.0） | 115（36.9） | 45（33.8） |  |
| First-line thrombectomy |  |  |  | 0.348+ |
| Stent-retriever, n (%) | 7（1.6） | 6（1.9） | 1（0.8） |  |
| Aspiration, n (%) | 44（9.9） | 27（8.7） | 17（12.8） |  |
| Stent-retriever plus aspiration, n (%) | 394（88.5） | 279（89.4） | 115（86.5） |  |
| Anesthesia, n (%) |  |  |  | 0.787 |
| General Anesthesia | 280（62.9） | 199（63.8） | 81（60.9） |  |
| Local Anesthesia | 136（30.6） | 94（30.1） | 42（31.6） |  |
| Sedation | 29（6.5） | 19（6.1） | 10（7.5） |  |
| IVT |  |  |  | 0.849+ |
| rt-PA, n (%) | 51（11.5） | 34（10.9） | 17（12.8） |  |
| TNK, n (%) | 7（1.6） | 6（1.9） | 1（0.8） |  |
| UK, n (%) | 4（0.9） | 3（1.0） | 1（0.8） |  |
| Types of Circulation |  |  |  | 0.542 |
| Anterior Circulation, n (%) | 234（52.6） | 167（53.5） | 67（50.4） |  |
| Posterior Circulation, n (%) | 211（47.4） | 145（46.5） | 66（49.6） |  |
| Way of rescue |  |  |  | 0.903 |
| Balloon, n (%) | 306（68.8） | 214（68.6） | 92（69.2） |  |
| Stent, n (%) | 139（31.2） | 98（31.4） | 41（30.8） |  |
| HbA1c, n (%), % | 5.9（5.5-7.0） | 5.9（5.5-7.1） | 5.9（5.6-6.7） | 0.831 |
| Tirofiban treatment, n (%) | 366（82.2） | 256（82.1） | 110（82.7） | 0.868 |
| Refractory thrombectomy, n (%) | 70（15.7） | 51（16.3） | 19（14.3） | 0.585 |
| Any ICH with 24hours, n (%) | 92（20.7） | 66（21.2） | 26（19.5） |  |
| SICH with 24 hours, n (%) | 20（4.5） | 16（5.1） | 4（3.0） |  |
| Vessel dissection, n (%) | 35（7.9） | 21（6.7） | 14（10.5） | 0.173 |
| Vascular perforation, n (%) | 3（0.7） | 2（0.6） | 1（0.8） | 1.000+ |
| Distal embolization, n (%) | 32（7.2） | 21（6.7） | 11（8.3） | 0.565 |
| Favorable outcome, n (%) | 188（42.2） | 132（42.3） | 56（42.1） | 0.968 |
| Thrombectomy year, n (%) |  |  |  | 0.093 |
| 2018 | 21 (4.7) | 18 (5.8) | 3 (2.3) |  |
| 2019 | 58 (13.0) | 37 (11.9) | 21 (15.8) |  |
| 2020 | 47 (10.6) | 36 (11.5) | 11 (8.3) |  |
| 2021 | 92 (20.7) | 67 (21.5) | 25 (18.8) |  |
| 2022 | 37 (8.3) | 31 (9.9) | 6 (4.5) |  |
| 2023 | 94 (21.1) | 62 (19.9) | 32 (24.1) |  |
| 2024 | 96 (21.6) | 61 (19.6) | 35 (26.3) |  |

+P, P value calculated by Fisher’s exact test.HT, hypertension; DM, diabetes mellitus; AF, atrial fibrillation; CHD, coronary heart disease; SBP, systolic blood pressure; DBP, diastolic blood pressure; MAP, mean arterial pressure; GLU, glucose; NIHSS, National Institutes of Health Stroke Scale; ASPECTS, Alberta Stroke Program Early CT Score; ODT, Onset-to-door time; OPT, Onset-to-puncture time; PRT, Puncture-to-recanalization time; DPT, Door-to-puncture time; LAA, large-artery atherosclerosis; CE, cardioembolism; ICA, internal carotid artery; rt-PA, recombinant tissue-type plasminogen activator; TNK, tenecteplase; UK, urokinase; HbA1c, glycated hemoglobin; Refractory thrombectomy, Number of passes ≥ 3 times.

Supplemental Table 2. Full logistic regression model with full-precision coefficients

| Variable | β | OR (95% CI) | P value | Unit |
| --- | --- | --- | --- | --- |
| Intercept | -3.739 | — | <0.001 | — |
| HbA1c | 0.2965 | 1.345 (1.14–1.61) | <0.001 | per 1% |
| NIHSS | 0.0806 | 1.084 (1.05–1.13) | <0.001 | per point |
| OPT | 0.000229 | 1.00023 (1.00008–1.00038) | 0.003 | per minute |
| PRT | 0.007669 | 1.008 (1.001–1.015) | 0.026 | per minute |
| Refractory thrombectomy | 0.6425 | 1.901 (0.92–4.09) | 0.091 | yes vs no |

OR, odds ratio; CI, confidence interval.

HbA1c, glycated hemoglobin; NIHSS, National Institutes of Health Stroke Scale; OPT, Onset-to-puncture time; PRT, Puncture-to-recanalization time; Refractory thrombectomy, Number of passes >3 times.

Supplemental Table 3. Full logistic regression model incorporating thrombectomy year in sensitivity analysis

| Variable | β | OR (95% CI) | P value | Unit |
| --- | --- | --- | --- | --- |
| Intercept | -4.249 | — | <0.001 | — |
| HbA1c | 0.2914 | 1.338 (1.14–1.60) | <0.001 | per 1% |
| NIHSS | 0.0812 | 1.085 (1.05–1.13) | <0.001 | per point |
| OPT | 0.000227 | 1.00023 (1.00008–1.00040) | 0.004 | per minute |
| PRT | 0.0101 | 1.010 (1.002–1.018) | 0.013 | per minute |
| Refractory thrombectomy | 0.6340 | 1.885 (0.91–4.06) | 0.096 | yes vs no |
| Thrombectomy year | 0.0929 | 1.097 (0.941–1.283) | 0.240 | per year |

OR, odds ratio; CI, confidence interval.

HbA1c, glycated hemoglobin; NIHSS, National Institutes of Health Stroke Scale; OPT, Onset-to-puncture time; PRT, Puncture-to-recanalization time; Refractory thrombectomy, Number of passes >3 times.

Supplemental Table 4. Comparison of model performance before and after incorporation of thrombectomy year

| Cohort | Model | AUC | P value for AUC comparison |
| --- | --- | --- | --- |
| Training cohort | Model 1 (without thrombectomy year) | 0.741 | 0.912 |
|  | Model 2 (with thrombectomy year) | 0.742 |  |
| Validation cohort | Model 1 (without thrombectomy year) | 0.781 | 0.552 |
|  | Model 2 (with thrombectomy year) | 0.766 |  |

AUC, area under the receiver operating characteristic curve; Model 1 comprised HbA1c, NIHSS, OPT, PRT, and refractory thrombectomy status; Model 2 incorporated all variables in Model 1 and additionally included thrombectomy year as a continuous variable.

Supplemental Fig. 1: Receiver operating characteristic (ROC) curve based on 10-fold cross-validation in the training cohort.

Supplemental Fig. 2: Enlarged view of the nomogram for clinically relevant ranges of onset-to-puncture time (OPT) and puncture-to-recanalization time (PRT).
